# A giant uterine tumor in a woman with myotonic dystrophy

**DOI:** 10.1002/ccr3.864

**Published:** 2017-02-23

**Authors:** Hiroshi Kataoka, Satoshi Ueno

**Affiliations:** ^1^ Department of NeurologyNara Medical UniversityKashiharaNaraJapan

**Keywords:** Dystrophia myotonica–protein kinase, leiomyoma, myotonic dystrophy

## Abstract

Patients with myotonic dystrophy are at particularly high risk for cancer arising in the endometrium, brain, colon, or ovary. Giant leiomyoma can occur in patients with myotonic muscular dystrophy, a disease accompanied by muscle wasting.

A 36‐year‐old woman presented with lower right abdominal pain. A giant uterine tumor was evident on radiographic, computed tomographic (CT), and magnetic resonance imaging (MRI) examinations (Fig. 1), and a total hysterectomy was performed. The resected uterus weighed 7.2 kg and measured 38 × 34 cm in diameter (Fig. 1). The histopathological diagnosis was leiomyoma. Because of syncope on postoperative day 1, a neurologist was consulted. No probable causes of syncope were evident on 12‐lead electrocardiography or cranial CT, and the results of laboratory examinations were normal, except for creatine kinase (781 U/L), glucose (141 mg/dL), lactate dehydrogenase (314 IU/L), C‐reactive protein (3.8 mg/dL), and white blood cells (11,000/*μ*L). Cervical bruit was absent. However, the patient had a myopathic face, muscular weakness of all four limbs and the neck, mild ptosis, and hypoactive deep tendon reflexes. Grip myotonia and percussion myotonia were present. Electromyography showed myopathic motor unit action potentials in the extremities, and typical myotonic discharges were recorded. A CTG expansion of 800 repeats in the dystrophia myotonica–protein kinase (DMPK) gene was confirmed. A potential cause of syncope was not found on echocardiography, 24‐h Holter monitoring, brain magnetic resonance angiography, or electroencephalography. This case is unusual because abdominal pain led to the detection of a giant leiomyoma as well as myotonic dystrophy.

**Figure 1:**
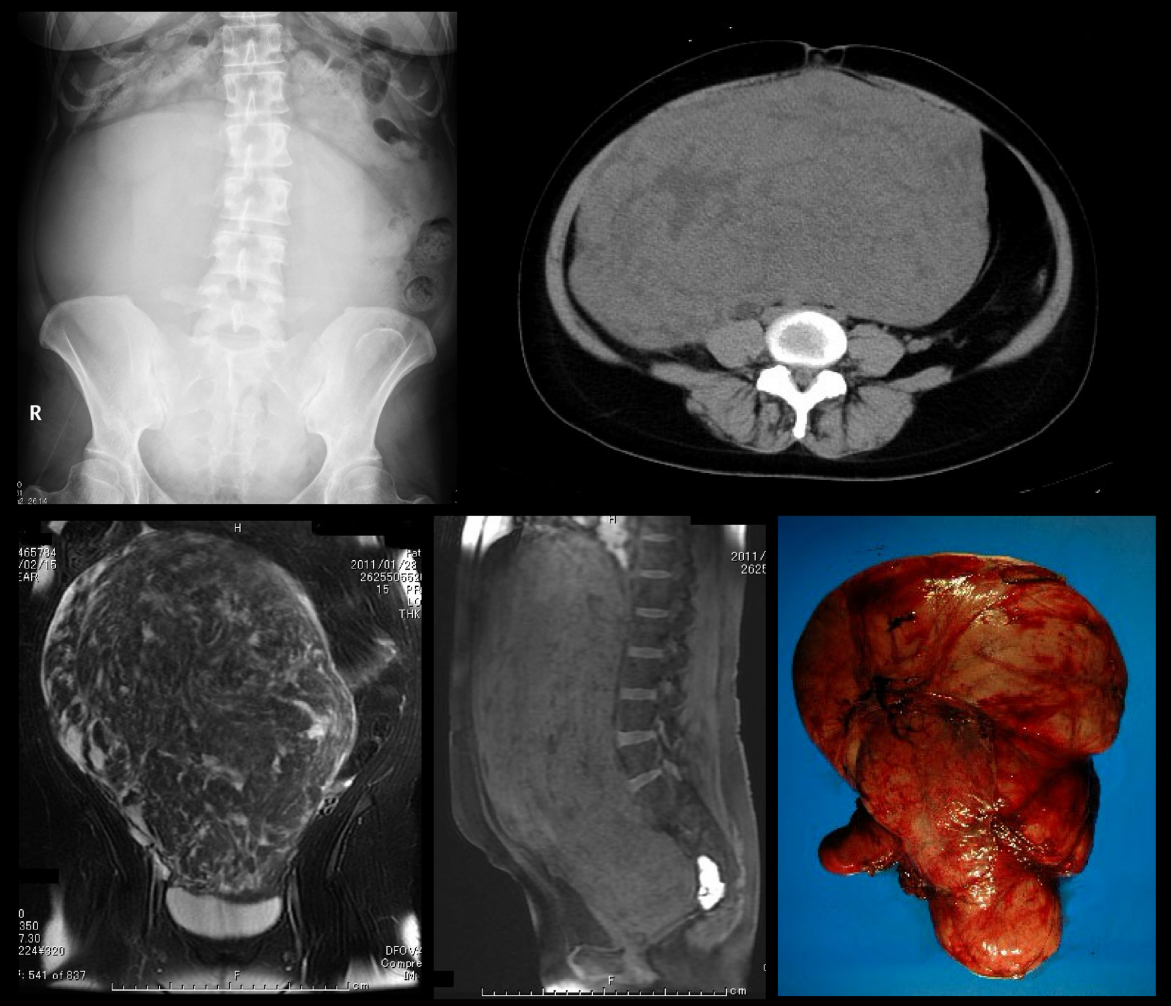
A giant uterine leiomyoma on an abdominal X‐ray film (upper left panel) and computed tomographic (upper right panel) and magnetic resonance images (lower left and middle panel). A macroscopic view is shown in the lower right panel.

Myotonic dystrophy can be associated with various tumors, but an association with myoma has not been reported previously 1. Epidemiological studies have provided evidence of an increased risk of certain types of malignant neoplasms, including melanoma and neoplasms of the thyroid, endometrium, brain, ovary, and colon, in patients with myotonic dystrophy 2. The DMPK gene has been suggested to act as a tumor‐suppressor gene in humans, and its impairment might contribute to the tumor growth found in patients with myotonic dystrophy 3.

## Consent

Written informed consent was obtained from the patient for publication of this case report and any accompanying images.

## Conflict of Interest

The authors declare that they have no competing interests.

## Authorship

HK: wrote the manuscript. HK: contributed to acquisition of data. HK: contributed to analysis and interpretation of data. HK and SU: contributed to drafting and critical revision of part of the submitted materials.
